# In vivo response to polypropylene following implantation in animal models: a review of biocompatibility

**DOI:** 10.1007/s00192-016-3029-1

**Published:** 2016-05-23

**Authors:** Michelle Kelly, Katherine Macdougall, Oluwafisayo Olabisi, Neil McGuire

**Affiliations:** Devices Division, The Medicines and Healthcare products Regulatory Agency (MHRA), 151 Buckingham Palace Road, London, SW1W 9SZ UK

**Keywords:** Biocompatibility, Host response, Mesh, Pelvic organ prolapse, Polypropylene, Stress urinary incontinence

## Abstract

**Introduction and hypothesis:**

Polypropylene is a material that is commonly used to treat pelvic floor conditions such as pelvic organ prolapse (POP) and stress urinary incontinence (SUI). Owing to the nature of complications experienced by some patients implanted with either incontinence or prolapse meshes, the biocompatibility of polypropylene has recently been questioned. This literature review considers the in vivo response to polypropylene following implantation in animal models. The specific areas explored in this review are material selection, impact of anatomical location, and the structure, weight and size of polypropylene mesh types.

**Methods:**

All relevant abstracts from original articles investigating the host response of mesh in vivo were reviewed. Papers were obtained and categorised into various mesh material types: polypropylene, polypropylene composites, and other synthetic and biologically derived mesh.

**Results:**

Polypropylene mesh fared well in comparison with other material types in terms of host response. It was found that a lightweight, large-pore mesh is the most appropriate structure.

**Conclusion:**

The evidence reviewed shows that polypropylene evokes a less inflammatory or similar host response when compared with other materials used in mesh devices.

**Electronic supplementary material:**

The online version of this article (doi:10.1007/s00192-016-3029-1) contains supplementary material, which is available to authorized users

## Introduction

Weakness of supporting tissues in the body, intended to maintain the integrity of bodily cavities, can result in the herniation of organs beyond their original location. This phenomenon can lead to altered function or damage to the organs and surrounding structures. Stress urinary incontinence (SUI) and pelvic organ prolapse (POP) are two common conditions that occur in the pelvic floor region [1]. POP occurs when loss of support of the vaginal wall causes one or more of the pelvic organs to protrude into or beyond the vagina [2–5]. Stress urinary incontinence is the involuntary leakage of urine upon increased intraabdominal pressure [2, 6].

To restore structural integrity and/or function various approaches have been used. Conservative methods are often attempted initially and, if unsuccessful, corrective surgery is an option. Traditional surgical techniques have included suturing local tissue to the Cooper’s ligaments to support the urethra in treating SUI and plication of the native tissue for POP repair [1–3]. These procedures can be associated with significant rates of recurrence of the original condition [7–13]. In an attempt to address this problem the use of synthetic materials were explored with incontinence and prolapse meshes, which are designed to provide a longer lasting outcome [3, 9, 14].

It should be noted that although meshes for the treatment of incontinence and prolapse have similarities in basic geometry, they are configured differently according to their intended use. The distinction between applications is important because reported complication rates differ significantly for these two device types [1, 15].

There is considerable heterogeneity among devices available for use in tissue defect repair in POP and SUI. It has therefore proven difficult for researchers to draw clear comparisons between these devices, and to identify which is the most appropriate material for specific applications [11, 16]. Mesh constructs are traditionally classified into four groups according to their pore size (see Table 1) [17]. This is a useful classification since the pore size of meshes has long been accepted as an important parameter for biocompatibility [18]. The significance of pore size can be seen when host responses to microorganisms are considered. The primary function of the immune system is to defend the body against invading pathogens. The cells involved in an immune response are greater in size than certain pathogens that cause infections; for example leukocytes average between 9–15 μm and macrophages 16–20 μm whereas bacteria average 2 μm in size. If the pore sizes in a mesh are less than that of relevant immune cells then bacteria present could remain in the mesh construct unchallenged thereby increasing the risk of infection to the patient. The spaces in some multifilament constructs, termed interstices, may act in a similar way to microporous mesh and therefore increase the risk of infection [17, 19, 20]. However, the utility of this classification system has begun to be questioned recently as mesh devices have continued to evolve. Nevertheless, there is as yet no clear consensus regarding an improved classification system [18].

**Table 1:** Amid’s classification of mesh constructs [17]

Type	Pore size
I	Completely macroporous: all pore sizes greater than 75 μm
II	Totally microporous: pore size smaller than 10 μm in at least one of the three dimensions
III	Macroporous with multifilamentous components
IV	Submicron pores

**Fig. 1:**
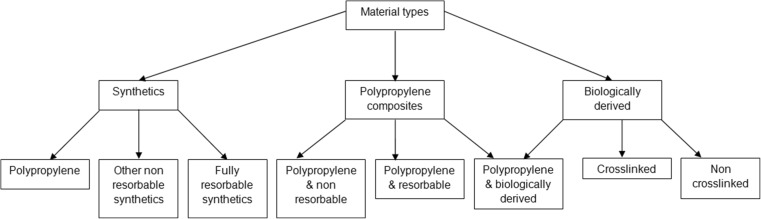
Summary of mesh types

A wide variety of materials have been used in medical devices for tissue repair, from synthetic to biologically derived, non-resorbable to resorbable, as demonstrated in Fig. 1 [1]. Of all the materials that are currently in use for mesh applications, polypropylene is the most common, and is the most firmly established [21, 22]. Polypropylene was first made in 1954 by Giulio Natta [23]. It is polymerised from propylene, an ethylene with one methyl group attached, such that in the polymer all the methyl groups face in the same direction, classing it as an isotactic polymer. Its superiority over other materials used in clinical mesh applications was realised by Francis C. Usher in 1962, partly because of its ability to be autoclaved [24]. In addition to the use of polypropylene in surgical meshes, this material can also be found in other common medical applications such as sutures [14]. Polypropylene is an obvious option for pelvic floor repair owing to its common use in abdominal wall and inguinal hernia repair [25, 26].

## Methods

A PubMed search for animal study articles published from January 1990 to September 2015 was conducted using the following search terms: “mesh”, “transvaginal”, “vaginal”, “pelvic region”, “pelvic floor”, pelvic organ prolapse”, “stress urinary incontinence”, “polypropylene”, “biomaterial”, “toxicology”, “biological safety”, “biocompatibility”, “in vivo”, “animal model”, “host response”. Additional relevant publications were also selected from the reference lists of the articles identified. Studies that primarily focused on in vitro implantation were excluded. Studies reporting on the biocompatibility or host response to incontinence or prolapse mesh were selected. Studies comparing treatments using polypropylene mesh with those using mesh of any other material, or where no mesh was used at all, were also selected. Non-comparative studies were excluded. A total of 585 different publications were identified in PubMed. Titles and abstracts were reviewed by three reviewers for relevance before obtaining the full paper. Forty-six papers were identified as being appropriate for inclusion in the study. References of these papers were then scanned and relevant papers were also included in the study. Selected studies were grouped together according to material type and anatomical location. Studies involving polypropylene were further subdivided according to mesh structure.

## Toxicology and biocompatibility

Owing to the nature of the complications experienced by some patients implanted with devices for SUI and POP, and the fact that polypropylene is the predominant material used, the toxicological properties and safety of this material have recently been called into question [27].

A biological risk assessment of any medical device, including incontinence and prolapse meshes, would have been conducted by the manufacturer based on laboratory testing before its first use in humans. This risk assessment would have included a review of all relevant toxicity endpoints in relation to the site and duration of exposure. Types of information used in this risk assessment include: consideration of the chemical nature of the materials, previous use in humans of the same or similar materials in comparable situations and, if required, results available from appropriate in vitro and/or in vivo biological tests, for example, those described in BS EN ISO 10993 series of standards to ensure that public health is not put at risk [28].

Data from clinical studies are an important consideration in any risk assessment, particularly those that address biological safety. However, none of the adverse effects that have been described with these procedures using polypropylene has been found to correlate directly with any toxicological property [29, 30]. To elucidate this, it is possible to compare this situation with that of another medical device type where a clinical outcome can be directly correlated with the toxicological properties of a particular material: aseptic loosening of joint replacement components. Joint replacements generate wear materials that the immune system identifies as foreign. This process results in bone loss and subsequent implant loosening, which may in turn require revision of the component. This process is termed osteolysis, and registry data have shown this to be the second most common reason for revision after infection [31]. Therefore, the extent of bone loss caused by the bearing materials can be explored using clinical studies. Because complications for SUI and POP cannot be linked to any one toxicological property, it is difficult to assess the toxicological properties of these devices using clinical studies.

Toxicology is the study of adverse effects of substances on living systems. This branch of science considers the inherent potential of xenobiotics to induce adverse health effects and whether under certain conditions of exposure that inherent toxic potential will translate into a risk to human health. Biocompatibility, often incorrectly used interchangeably with toxicology, can only be demonstrated under a defined set of circumstances [28]. Biocompatibility has been defined as “the ability of a material to perform with an appropriate host response in a specific application” by Williams in 1987 and is still a well-accepted definition today [32]. Biocompatibility is dependent on a number of factors, including inter-individual variations in tissue responses and factors related to procedure. It is important to appreciate that it is possible for a device to display a suitable response in one patient but not in another, for example, capsular contracture, a complication reported in some women who have received breast implants [28].

Any implanted material will evoke a host response to the foreign body, often termed foreign body reaction, and this is dependent upon patient susceptibility. This phenomenon is unavoidable and is characterised by seven interrelated stages post-implantation: injury, protein adsorption, acute inflammation, chronic inflammation, foreign body reaction, granulation tissue formation and tissue encapsulation [33].

The authors’ initial review of the literature regarding pelvic floor constructs concluded that host response was the main consideration rather than inherent toxicity of materials. Animal studies are useful for understanding the adverse effects directly related to the material. They enable standardisation of experimental parameters such as environmental conditions, material exposure and technique [28]. Therefore, this review focuses on the literature relating to the in vivo response to polypropylene and other materials used in incontinence and prolapse mesh applications when implanted in animal models.

## Material discussion

### Non-resorbable synthetic materials

Any foreign material implanted in the body has the potential to elicit a host response. The immune responses mounted by different individuals can differ significantly, despite the same materials and surgical techniques having been used [34]. This section discusses in vivo studies comparing polypropylene with three other synthetic materials used in tissue repair: polytetrafluoroethylene (PTFE), polyester and polyethylene terephthalate. Polypropylene is widely reported to be more biocompatible and has been shown to elicit the lowest level of inflammatory response compared with other polymeric materials such as nylon, polyacrylonitrile and polyethylene terephthalate [35, 36].

A review of the literature has shown that the host response to polypropylene is comparable with, or better than, the response to polyester-, PTFE- and expanded PTFE-based meshes in animal models. Novotny et al. found that polypropylene was comparable with PTFE in terms of biocompatibility when a sample of mesh was implanted intraperitoneally in New Zealand white rabbits. At 90 days post-implantation, the number of inflammatory cells and granuloma formation were seen to be similar [37]. All other studies reviewed by this group found comparable results. Hengirmen et al. reported that PTFE elicited a more pronounced foreign body reaction compared with polypropylene 12-week post-implantation in a rat abdominal wall. The authors concluded that polypropylene is a more suitable mesh material than PTFE for abdominal repair [38]. This finding was supported by Harrell et al. when expanded PTFE mesh was compared with polypropylene in a rabbit model following implantation over a longer period of time [39]. Bleichrodt et al. took this one stage further and measured the outcome of both these mesh materials when implanted in a contaminated environment in a rat model. The surgical site was contaminated using a rat faeces suspension 1 week before implantation of the mesh under investigation. It was found that hernia recurrence was significantly greater in the expanded PTFE group compared with the polypropylene group. The authors concluded that expanded PTFE is unsuitable for use in the repair of infected abdominal walls [40]. This is a potentially important finding when selecting a material suitable for use in the pelvic floor region, as it has been widely reported that there is an increased risk of infection when materials are introduced via the vaginal route [41, 42].

Polypropylene-based meshes also fared well when compared with polyester-based meshes. Zinther et al. found that polyester elicited a more pronounced host response than polypropylene meshes, coated with collagen and polyvinylidene fluoride respectively, in the peritoneum of sheep 2 years after implantation [43]. The authors also found that where polyester meshes were implanted there was greater shrinkage than was observed with polypropylene [43]. Orenstein et al. compared polypropylene with both polyester and expanded PTFE meshes implanted in a mouse model. Similar to the findings above, the authors found that polypropylene induced a less pronounced foreign body reaction compared with the other two materials and polyester performed less well with regard to biocompatibility, showing a more marked foreign body reaction. Foreign body reaction in this study was measured in terms of the number of foreign body giant cells around the mesh fibres [44].

Boulanger et al. compared polypropylene mesh with another synthetic polymer, polyethylene terephthalate, fixed onto the peritoneum of female pigs [45]. The polyethylene terephthalate mesh generated a more intense inflammatory reaction compared with polypropylene-based meshes in terms of higher macrophage and lymphocyte cell count 72 days post-implantation. Tissue integration was also less favourable in terms of the presence of collagen fibre organisation, fibroblasts, and vascularisation [45].

### Polypropylene composites

In an attempt to modify host responses to synthetic meshes, a range of composite materials have been examined [1]. Three classes of new composite material meshes, all incorporating polypropylene, are reviewed in this section: polypropylene with expanded PTFE, polypropylene with a resorbable constituent and polypropylene with a biologically derived component.

Expanded PTFE was selected as an additional biomaterial thought to reduce inflammation and impede undesirable tissue ingrowth that could lead to adhesion formation owing to its microporous structure [46]. Marcondes et al. and Harrell et al. found that polypropylene mesh was comparable with a composite of polypropylene with expanded PTFE in terms of foreign body reaction in rabbit models [25, 39, 48]. Marcondes et al. determined using histological analyses that the inflammatory reactions in the two groups were similar 20 days after implantation [47]. As indicated above, a comparable result was reported by Harrell et al. 4 months after implantation in terms of inflammatory cell count and tissue in-growth, despite this study using one of the earliest meshes, a heavyweight mesh [39].

Resorbable components have begun to be incorporated into constructs in an attempt to improve the handling characteristics of polypropylene mesh [25, 39, 48]. Polypropylene mesh with a resorbable component was not found to improve biocompatibility compared with polypropylene when implanted in the abdomen and bladder of a rat model and the abdomen of rabbit and pig models [46, 47, 49–53]. In a recent study conducted by Utiyama et al., polypropylene mesh was compared with polypropylene with a resorbable polyglecaprone film implanted into the abdomen of Wistar rats. No significant differences in the host’s response were identified in terms of the percentage of fibrosis, adhesions, shrinkage and various inflammatory cell counts [53]. Boulanger et al. noted that a partially resorbable mesh with a polyglactin component elicited an intense inflammatory reaction when implanted onto the peritoneum of pigs compared with polypropylene meshes [45]. Discussions on whether this composite fulfils all other essential requirements for use as a mesh is outside the scope of this review.

Another variant that has been investigated is the use of biologically derived substances as a coating on polypropylene mesh thought to mitigate the host’s foreign body response. This in turn would improve in-growth and reduce erosion and mesh exposure [54]. Similar to the findings above, the testing of these components in vivo has found that this technology does not offer an improvement in biocompatibility compared with polypropylene. Huffaker et al. found that polypropylene mesh coated with porcine collagen elicited a similar mild foreign body reaction and low-grade fibrotic response to uncoated polypropylene when implanted into the vagina of New Zealand white rabbits for 12 weeks [55]. A similar result was achieved for both materials by Pierce et al. 90 days after implantation in the abdominal wall of rats [56]. Van’t Riet et al. found that polypropylene was less susceptible to infection than collagen-coated polypropylene mesh following implantation in the abdominal wall of rats for 30 days [57]. Despite advances in these composite materials, the above findings demonstrate that polypropylene remains comparable with or superior to polypropylene composite mesh with regard to host responses when implanted in experimental animal models.

### Biologically derived meshes

The use of biologically derived materials has been investigated in the last decade with the aim of reducing complications associated with pelvic floor repair, such as erosion and dyspareunia [58]. Biologically derived materials are classed as acellular collagen matrices and are often derived from the dermis, pericardium, small intestinal submucosa or urinary bladder matrix of bovine or porcine origin [1, 22, 59]. These materials can undergo crosslinking, the formation of excessive intra- and inter-molecular bonds, to improve the longevity of the material [59]. Despite their use in other medical device applications, previously published work has concluded that non-crosslinked biological matrices are inappropriate because of their rapid degradation in situ [1, 59, 60]. In contrast, they found that crosslinked matrices may be more appropriate owing to a longer degradation profile, despite the increased risk of encapsulation [1, 59, 60]. However more evidence is needed to support these findings. Polypropylene mesh was found to have a longer-lasting and more supportive structure compared with porcine-derived matrices implanted in the abdomen of rats and rabbits, attributed to the fibrosis associated with the normal foreign body response to polypropylene [38, 61]. Krambeck et al. also found that polypropylene induced less inflammation and eosinophil infiltrate after 12 weeks when implanted in rabbits [61]. In addition to these findings, longer term studies in a rabbit model have also shown polypropylene in a favourable light compared with an acellular collagen mesh. Christodoulou et al. found that polypropylene had superior mechanical properties when mesh was implanted for 9 months in the rabbit abdomen [62]. Despite using a crosslinked material, Pierce et al. found that crosslinked porcine dermis degraded after 9 months following implantation in both the vagina and abdomen of rabbits [58]. This degradation was accompanied by a greater, and more intense, inflammatory reaction than polypropylene. This study also investigated the rate of erosion. Erosion has been defined as the superficial destruction of a surface by friction, pressure, ulceration, or trauma [19]. The authors reported a higher erosion rate in the polypropylene group (27 %) compared with crosslinked porcine dermis (15 %) [58]. A similar finding was reported by Fan et al., who found that crosslinked urinary bladder matrix was associated with less erosion compared with polypropylene after 12 weeks’ implantation in the rabbit vagina [22]. In both studies no erosion was found when these materials were implanted in the abdomen of rabbits [22, 58]. In contrast to the above studies, polypropylene mesh fared well in terms of graft-related complications compared with crosslinked acellular collagen matrix derived from bovine pericardium implanted into the vaginal region of sheep for 180 days. Lower rates of contractility and calcification were observed with polypropylene mesh [60]. In conclusion, analyses of the literature reports available have revealed that polypropylene is more appropriate in terms of the mechanical requirements of pelvic floor repair. Over time, the biologically derived materials discussed above are prone to degradation, leaving behind a weak structure that increases the risk of recurrence when used for tissue repair. Overall, polypropylene has also fared well in terms of host response compared with several types of biologically derived materials. Recently Shi et al. investigated the biocompatibility of decellularised human amniotic membrane as a potential mesh material, with favourable results [63]. However further studies would need to be carried out to investigate if this is a viable option.

### Fully resorbable meshes

All foreign materials are associated with a chronic inflammatory reaction, which is variable depending on specific factors, including patient characteristics. Therefore, implanting foreign materials will never be completely risk free. A material that degrades over time is not associated with the same level of risk as the inflammatory reaction would normally be expected to resolve once complete degradation had occurred. The current view is that the only material type that truly meets the definition of biocompatibility is one that resorbs fully over time [1].

Mesh-related complications due to host response issues have proven particularly difficult to manage in certain patients. This has led to research into the feasibility of using fully resorbable meshes for tissue repair [64]. The use of this technology has been explored as far back as the 1980s, with initially poor results for long-term mechanical support and the resultant high recurrence rates [65]. Resorbable meshes have been shown to have a comparable acute inflammatory response to polypropylene [45]. However, none of the types of resorbable meshes investigated have resulted in adequate tissue incorporation. As a result of this, resorbable meshes were deemed unsatisfactory when prolonged tensile strength is required, such as that needed for the treatment of SUI and POP [53, 65, 66]. Recent improvements have been reported by Hjort et al. using a novel resorbable material, namely a copolymer of glycolide, lactide and trimethylene. Following implantation in the abdomen of a sheep model, strong connective tissue was observed after 36 months, with none of the animals showing recurrence at the end of the study. The authors stated that this is the world’s first long-term resorbable mesh and takes a minimum of 6 months to degrade, thereby allowing sufficient time for a collagen matrix to form [67]. Similarly, de Tayrac et al. demonstrated that the use of polylactic acid (PLA) could be a promising alternative material for long-lasting pelvic floor repair because of its slower degradation rate, which is attributed to the small fibres. A 12-month in vitro test showed that PLA mesh maintains a reduction of 30 % in its mechanical strength for the first 6 months [64]. In addition to this, PLA mesh was shown to have comparable mechanical properties with polypropylene mesh 3 months after implantation in rats [68]. However, these promising findings would need to be supported by long-term in vivo testing [69].

Further investigation into the biocompatibility of resorbable meshes is required to validate the findings above and to understand fully the prospect of using resorbable materials in tissue repair applications that require mechanical support.

## Structure and size of mesh

### Pore size and weight

As discussed above, the in vivo responses to polypropylene have been shown to be at least comparable with all other materials used in mesh applications. In view of this, the variations in the structure of polypropylene meshes have been investigated to determine if there are any biological response differences. The effects of pore size and weight have been analysed following implantation in rodent, pig and rabbit models. Studies in the literature reveal that the lighter the polypropylene mesh per square metre, the less pronounced the foreign body response observed [19]. A similar outcome was observed when the pore size was increased across the mesh. These effects have been observed when several biocompatibility endpoints were measured, namely: granuloma formation, scar bridging formation, in addition to inflammatory cell and mediator expression [18, 39, 44, 52, 70–72]. The impact of pore size on scar formation was investigated by Klinge et al. when implanting polypropylene mesh in the abdomen of rats for 90 days. A heavy-weight, small-pore mesh was associated with intense chronic inflammation accompanied by extensive scar bridging. Conversely, the lower-weight, large-pore mesh exhibited scarring similar to the control group. The authors state that pore size is a significant factor in the tissue’s response and the overall biocompatibility of polypropylene mesh [18].

Feola et al. found a difference in the biomechanical properties of the vagina when implanting meshes of different weights in nonhuman primates. The most pronounced adverse outcome was observed following implantation of the heaviest polypropylene mesh. The heaviest mesh had the greatest negative impact on both active and passive mechanical properties, where a greater reduction in vaginal contractility and tissue stiffness of the vagina was found [73]. This is thought to be due to the effect of stress shielding, a phenomenon that occurs when the stiffer material blocks or reduces the adjacent material from the full impact of physiological loading [73, 74]. This idea is supported in a study by Liang et al., who also implanted meshes of different weights in nonhuman primates. The heaviest polypropylene mesh induced the highest level of matrix-degrading enzymes compared with the lighter polypropylene meshes [75].

There is a lack of evidence relating to the mechanical requirements for a prolapse repair. However, it is accepted that the requirements are greater than that necessary for an abdominal defect repair. Understanding these requirements fully is crucial in determining how far it is possible to reduce the weight of a material and for it still to perform its necessary functions. Work carried out by Cobb et al. pointed to the feasibility of reducing the weight of a mesh beyond that currently considered to be a light-weight construct. It has been found that the lightest mesh tested still far exceeded the burst strength of native abdominal wall tissue when implanted into a porcine ventral hernia model. Furthermore, there was no significant difference in the mean burst loads between the medium (590 N) and lightweight (576 N) meshes. The fact that the burst load for the heavyweight mesh (1,200 N) was much greater, suggests a plateau effect between the mid- and lightweight meshes [76]. This indicates that it may be possible to further reduce the weight of a pelvic floor construct. However, caution must be exercised, as demonstrated by Ozog et al., who attempted to implant an ultra-lightweight polypropylene mesh of 7.6 g/m^2^ into the abdomen of rabbits. The authors deemed the handling characteristics of this mesh inappropriate because of folding upon insertion, which was not observed with the heavier mesh [77].

### Monofilament versus multifilament

In addition to the above observations, Krause et al. investigated differences in biological responses between type I (monofilament) and type III (monofilament with a multifilamentous component) meshes. The authors found that when a type III mesh was implanted into the abdominal wall of rats for 3 months the resultant inflammatory and fibrous reactions were more pronounced and persistent compared with those of type I mesh [78]. This is thought to be due to the interstices created in multifilament constructs, which is an important consideration because, as already discussed, small pores are thought to increase the likelihood of bacteria being inaccessible to immunocompetent cells. This was demonstrated by Díaz-Godoy et al. when polypropylene meshes of varying pore sizes were tested in a contaminated rabbit model. It was found that the larger the pore size, the fewer the number of animals that were found to have infection at the end of the study. In fact, none of the animals implanted with the meshes with the largest pore size displayed clinical signs of infection, or positive biological cultures on the last day of the study [79].

The potential clinical relevance to humans of the above findings was demonstrated by Badiou et al. when cases of erosion were found to be significantly higher in animals showing signs of infection, in terms of increased bacteria count around the mesh, compared with animals without infection. These comparative studies were conducted using mesh that was sutured to the surface of the abdominal wall of female Wistar rats. Following closure of the surgical site a suspension of *Escherichia coli* was injected around the mesh [80]. This supports the current view that infection is a significant risk factor in the development of erosion [19].

### Size of mesh

Pierce et al. found that the erosion rate was significantly reduced when smaller pieces of graft material were used. The rate decreased from 27 % to 10 % for smaller polypropylene mesh when implanted in the vagina of rabbits and analysed after 9 months [58]. This finding was replicated in a sheep model by Manodoro et al., where it was reported that polypropylene mesh, when reduced in area from 50 mm^2^ to 35 mm^2^, resulted in a decrease in erosion rate from 30 % to 0 % when implanted in the vagina [81]. These findings suggest that, all other factors being equal, the amount of material might be directly proportional to the erosion rate, a known complication in the presence of an intense foreign body response [19, 77].

## The impact of anatomical location

To explore whether differences exist between the placement of mesh in the abdominal and the vaginal regions, investigators have examined the impact of anatomical location on host responses using the New Zealand white female breeder rabbit. This animal is seen as a more suitable model than rodents when conducting vaginal surgery research [22]. A few studies have shown that the placement of mesh in the vaginal region results in a more pronounced inflammatory reaction compared with abdominal placement [22, 58, 82]. Fan et al. observed an erosion rate of 67 % and evidence of necrosis when polypropylene mesh was placed in the vagina of rabbits for 12 weeks, compared with no erosion observed when placed in the abdomen. However, the same authors advise caution when interpreting these results, as there was difficulty modelling the vagina because of the small operating space, an issue not experienced in abdominal surgery modelling [82]. In support of this, Huffaker et al. found that no erosion occurred following implantation of lightweight monofilament polypropylene meshes in the vagina of New Zealand white rabbits after 12 weeks [55]. In a more recent study investigating host response differences between the vagina and abdomen, Endo et al. implanted biologically derived mesh into sheep for 180 days. It was found that a greater number of graft-related complications were observed with mesh at the vaginal site compared with the abdominal site. In particular, the contraction rate was three times higher in the vagina than in the abdomen. There was also a marked difference in the occurrence of degradation between the groups. Degradation of the acellular collagen matrix coincided with an abundance of foreign body giant cells. It is not yet known if this degradation process is a faster or more vigorous process at the vagina [59].

Despite the paucity of studies in this area, the current findings discussed above suggest that the pelvic region might be more susceptible to complications than the abdominal region in relation to heightened host response. The potential clinical relevance is not yet understood. The very high erosion rate reported in the study by Fan et al. is not typically observed in humans and this could indicate interspecies differences [22].

## Conclusion

Pelvic organ prolapse and stress urinary incontinence are two pelvic floor disorders that are often treated with the use of synthetic materials. One of the first materials used in the construction of mesh devices, and one that is still predominantly used today, is polypropylene. Other materials have also been evaluated for their use in mesh applications ranging from alternative synthetic to biologically derived materials. The use of resorbable components has also been explored, either as an addition to the polypropylene or as a fully resorbable device.

This review has investigated the suitability of polypropylene as an implantable material by comparing the in vivo host responses of polypropylene in animals with other material types. This has revealed that polypropylene evokes a less inflammatory or similar host response compared with other synthetic materials and polypropylene composite meshes. This is a useful finding, as animal studies are a good indicator of how a material may behave in humans. Promising biocompatibility outcomes have been observed with the use of biologically derived and fully resorbable meshes. However, both these types of material currently lack the mechanical strength required for long-lasting repair, thereby increasing the risk of recurrence of the original problem.

Selecting the most appropriate material for a given procedure requires a risk-based analysis to be conducted by surgical staff. This should include consideration of the likelihood of recurrence and the risk of complications.

A review of the structural differences within polypropylene meshes points favourably towards using a light-weight mesh with large pores. Small pore sizes have been associated with a higher infection rate, attributed to the difference in size between immune cells and pathogens. This outcome is thought to be significant as infection is a factor that increases the risk of erosion, a known complication with mesh devices. In addition to this, lighter-weight, more porous mesh allows for more normal loading on the surrounding tissue, leading to improved tissue in-growth.

It is known that the surface area of material implanted is proportional to the host response produced. The cause of this is multifactorial and includes patient and material factors. The impact of anatomical location has only been researched by a small number of authors. Despite this, the current evidence suggests that the pelvic region is more susceptible to exhibiting host response issues than the abdominal region. However, anatomical location and size of mesh are often factors that cannot be adjusted when repairing a prolapse in the pelvic floor region, which may in turn lead to a higher level of complications.

The available evidence suggests that there might be scope for reducing the weight of the mesh beyond that currently considered to be light-weight, yet retaining the desired mechanical properties. This may contribute to reducing the amount of material against which a host response could be mounted and the level of inflammation. However, given the multifactorial nature of complications, the overall impact of doing this may currently be difficult to determine.

Further work is required to understand the differences in host response between mesh device placements in the pelvic compared with the abdominal region. Interspecies differences will need to be explored in greater detail in an attempt to find the most suitable animal model.

## Electronic supplementary material

Below is the link to the electronic supplementary material.

ESM 1 (DOC 127 kb)
