# Supplementary material for: In vivo response to polypropylene following implantation in animal models: a review of biocompatibility
Source: Int Urogynecol J. 2016 May 23;28(2):171–80. doi: 10.1007/s00192-016-3029-1 (PMC5306078; doi:10.1007/s00192-016-3029-1)
Supplement: Supplementary file 1 — (DOC 127 kb) [file 192_2016_3029_MOESM1_ESM.doc]

| **Study** | **Animal model** | **Location of implantation** | **Sample size** | **Materials compared** | **Host response** |
| --- | --- | --- | --- | --- | --- |
| ***Non-resorbable synthetic materials*** | | | | | |
| **Usher et al. (1962)** | Dog | Abdomen | 18 | - Polypropylene - Nylon - Polyacrylonitrile - Polyethylene terephthalate - PTFE | Polypropylene had low foreign-body reaction, considerably less than that observed with nylon, polyacrylonitrile and polyethylene terephthalate and PTFE. |
| **Novotný et al (2012).** | Rabbit | Abdomen | 14 | - PTFE - Polypropylene | Biocompatibility of PTFE mesh comparable to light-weight polypropylene. |
| **Hengirmen et al (1998).** | Rat | Abdomen | 45 | - PTFE - Porcine dermal collagen - Polypropylene | PTFE showed more intense foreign body reaction compared to polypropylene. |
| **Harrell et al (2007).** | Rabbit | Abdomen | 40 | - Expanded PTFE - Expanded PTFE and polypropylene composite - Polypropylene and oxidized regenerated cellulose composite - Polypropylene | All materials demonstrated comparable host biocompatabitly. Inflammatory cell count greater around expanded PTFE compared to mid-weight polypropylene. |
| **Bleichrodt et al (1993).** | Rat | Abdomen | 42 | - Expanded PTFE - Polypropylene | Incisional hernia and fistula formation higher in expanded PTFE group. |
| **Zinther et al (2010).** | Sheep | Abdomen | 16 | - Polyester - Polypropylene | Increased foreign body reaction and shrinkage with the polyester based mesh. |
| **Orenstein et al (2012).** | Mouse | Abdomen | 30 | - Polyester - Expanded PTFE - Polypropylene | Polyester mesh greatest inflammatory response and marked foreign body reaction. Expanded PTFE moderate foreign body reaction with mesh encapsulation at 12 weeks. All polypropylene meshes had lowest foreign body reaction. |
| **Boulanger et al (2006).** | Pig | Abdomen | 12 | - Polyglactin 910 - Polypropylene - Polypropylene and polyglactin 910 composite - Polyethylene terephthalate | Polypropylene meshes had low level of inflammation in contrast to polyethylene terephthalate mesh.  Polypropylene mesh had more favourable tissue integration with well-organised fibrous, mature, connective tissue. |
| ***Polypropylene composites*** | | | | | |
| **Byrd et al. (2011)** | Pig | Abdomen | 16 | - Polypropylene/expanded PTFE composite mesh with a novel internal polydioxanone absorbable ring. | This composite mesh was associated with minimal intraabdominal adhesions, progressive in-growth of host tissues, and complete degradation of a novel internal PDO ring that aided mesh positioning. This composite hernia mesh showed a favourable performance in a porcine model of open ventral hernia repair. |
| **Marcondes et al (2008).** | Rabbit | Abdomen | 24 | - Polypropylene - Polypropylene and sodium hyaluronate-carboxymethylcellulose - Polypropylene and expanded polytetrafluoroethylene | Polypropylene mesh was comparable to a composite of polypropylene with expanded PTFE in terms of inflammation. Polypropylene and sodium hyaluronate-carboxymethylcellulose mesh induced a higher inflammatory reaction compared with the other meshes. |
| **Junge et al (2005).** | Rat | Inguinal ligament | 30 | - Polypropylene - Polypropylene and poliglecaprone 25 composite | Both mesh materials showed a slight foreign body reaction with no significant difference. |
| **Quinino et al (2013).** | Rabbit | Abdomen | 20 | - Polypropylene - Polypropylene with Coseal® spray | No differences in the inflammatory response, fibrosis, foreign body reaction, presence of collagen and type of inflammatory cells between the two groups; polypropylene mesh and polypropylene mesh coated with a reabsorbable hydrogel of polyethylene glycol. |
| **Vogels et al (2015).** | Rat | Abdomen | 80 | - Condensed PTFE - Polypropylene and poliglecaprone 25 composite - Polyester and polyurethane composite - Polypropylene | No meshes were found to be superior in all aspects required for effective and safe incisional hernia repair. |
| **Scheidbach et al (2004).** | Pig | Groin | 44 | - Heavy weight polypropylene - Light weight Polypropylene - Titanium-coated light weight polypropylene - Light weight polypropylene and polyglactic acid composite | More pronounced inflammatory reaction and greater shrinkage observed with polypropylene and resorbable composite mesh. |
| **Murat et al (2004).** | Rat | Bladder | 48 | - Polypropylene - Polyglactin - Polypropylene and polyglactin composite | Tissue reactions to polypropylene and polyglactin composite meshes were more inflammatory at 7 days but the tissue reactions to all mesh materials were similar at 14 days. |
| **Utiyama et al (2015).** | Rat | Abdomen | 30 | - High density Polypropylene - Low density Polypropylene and polyglecaprone composite | No significant difference was found between high-density or low-density polypropylene meshes at 21 days after surgery in terms of inflammatory cell infiltrate, mesh shrinkage, occurrence of adhesions and complications. |
| **Boulanger et al (2006).** | Pig | Abdomen | 12 | - Polyglactin 910 - Polypropylene - Polypropylene and polyglactin 910 composite - Polyethylene terephthalate | The absorbable prostheses (polyglactin 910) and the non-absorbable prostheses (polypropylene) induced the least severe inflammatory response.  Polypropylene meshes had a low level of inflammation in contrast to polyethylene terephthalate mesh. |
| **Huffaker et al (2008).** | Rabbit | Vagina | 10 | - Porcine collagen-coated polypropylene - Polypropylene | Both coated and uncoated polypropylene meshes elicit a mild foreign body reaction and minimal fibrotic response without evidence of vaginal epithelial erosion. |
| **Pierce et al (2011)** | Rat | Abdomen | 54 | - Porcine collagen coated polypropylene - Uncoated polypropylene | Collagen-coated polypropylene mesh induced elevated inflammatory reaction compared with uncoated mesh during the early post-operative period (<90 days). |
| **Van't Riet et al (2004).** | Rat | Abdomen | 58 | - Polypropylene - Polypropylene with collagen coating | Collagen-coated polypropylene mesh was more susceptible to mesh infection than polypropylene mesh. Comparable mesh incorporation in the abdominal wall with both mesh types. |
| ***Biologically derived meshes*** | | | | | |
| **Pierce et al (2009).** | Rabbit | Vagina and abdomen | 45 | - Polypropylene - Cross-linked porcine dermis | Polypropylene induced a milder, more uniform response than cross-linked porcine dermis, whereas cross-linked porcine dermis elicited a more variable response and degraded by 9 months. Vaginal grafts had higher scores for inflammation and neovascularization and had lower scores for fibroblastic proliferation than abdominal grafts. |
| **Fan et al (2014).** | Rabbit | Vagina and abdomen | 40 | - Polypropylene - Porcine-derived, cross-linked urinary bladder matrix | Vaginal polypropylene mesh was associated with an erosion reaction, whereas abdominal polypropylene mesh and cross-linked urinary bladder matrix showed no sign of erosion.. Compared with vaginal polypropylene mesh, vaginal cross-linked urinary bladder matrix induced milder chronic inflammation response, had lower scores for inflammation response, and showed higher scores for neovascularization and fibroblastic proliferation. In the abdomen, both histopathological parameters were insignificantly different between polypropylene mesh and cross-linked urinary bladder matrix. |
| **Endo et al (2015).** | Sheep | Vagina and abdomen | 10 | - Cross-linked acellular collagen matrix - Polypropylene | More graft related complications observed with mesh at the vaginal site compared to the abdominal site. Contraction rate three times higher in the vagina compared to the abdomen. |
| **Butler et al (2010)** | Guinea - pig | Abdomen | 58 | - Non-cross-linked porcine acellular dermal matrix - Cross-linked porcine acellular dermal matrix | Non-cross-linked porcine acellular dermal matrix rapidly infiltrated with host cells and vessels; cross-linked PADM becomes encapsulated. |
| **Krambeck et al (2006).** | Rabbit | Rectus fascia | 10 | - Human cadaveric fascia - Porcine dermis - Porcine small intestine submucosa - Polypropylene mesh - Autologous fascia | Polypropylene mesh had lowest inflammatory reaction in terms of eosinophil infiltrate, and inflammatory rind.  Polypropylene mesh, cadaveric fascia, and porcine dermis all demonstrated a high presence of fibrosis.  Polypropylene mesh had greatest degree of scar formation at 12 weeks, thought to contribute to a more lasting repair. |
| **Christodoulou et al (2013).** | Rabbit | Rectus abdominus | 96 | - Acellular collagen mesh - Polypropylene | Tissue with polypropylene mesh showed greatest dynamic rigidity. Those with biological mesh delivered the lowest rigidity results, while the no mesh groups had almost similar behaviour. |
| **Shi et al (2015).** | Rabbit | Uterus | 2 | - Human amniotic membrane/ poly(ester urethane) composite - Polypropylene | Decellularized human amniotic membrane possessed good biocompatibility in terms of inflammation and tissue remodelling compared to polypropylene. |
| ***Fully resorbable meshes*** | | | | | |
| **De Tayrac (2008).** | Rat | Abdomen | 15 | - poly(L-lactic acid) | Poly(L-lactic acid) caused a mild inﬂammatory response in animals and was biocompatible at short-term in an animal model of incisional hernia. |
| **Lamb et al (1983).** | Rabbit | Abdomen | 15 | - Polytetrafluoroethylene - Polyglactin - Polypropylene | At 3 weeks polyglactin mesh had a bursting strength comparable to that of control flaps but at 12 weeks was significantly weaker. Fibrous tissue incorporation within the mesh fibres was better with PTFE mesh than with polypropylene mesh. |
| **Tyrell et al (1989).** | Rabbits | Abdomen | 80 | - Polypropylene - Polytetrafluoroethylene - Polyglactin - Polyglycolic acid | No hernias were observed with the nonabsorbable meshes, but all of the rabbits repaired with absorbable meshes had ventral hernias by the tenth week. |
| **Boulanger et al (2006).** | Pig | Adbomen | 12 | - Polyglactin 910 - Polypropylene - Polypropylene and polyglactin 910 composite - Polyethylene terephthalate | The absorbable prostheses (polyglactin 910) and the non-absorbable prostheses (polypropylene) induced the least severe inflammatory.  The polypropylene mesh had more favourable tissue integration with well-organised fibrous, mature, connective tissue. |
| **Hjort et al (2012).** | Sheep | Abdomen | 14 | - Polypropylene - Glycolide, lactide and trimethylene copolymer | Resorbable mesh characterized by a collagen deposition more similar to native connective tissue and an increased thickness of the integrating tissue. Polypropylene mesh provoked a typical chronic inflammation persistent over the 36-month study period.  . |
| **De Tayrac et al (2007).** | Rat | Abdomen | 45 | - Poly(lactic acid) - Polypropylene - Poly(glycolic acid) | Poly(lactic acid) mesh induced a milder inflammatory response, more orderly collagen deposition than polypropylene, and preserved comparable tensile strength after 90 days. |
| **De Tayrac et al (2010).** | Rat | Abdomen | 108 | - Poly(lactic acid) - Polypropylene | Poly(lactic acid) mesh induced an inflammatory response significantly less than that of polypropylene mesh.  Sterilisation of poly(lactic acid) reduced the tensile strength and herniation occurred. No herniation occurred in the polypropylene group. |
| ***Pore size and weight*** | | | | | |
| **Klinge et al (2002)** | Rat | Abdomen | 75 | - Heavyweight polypropylene mesh - Lightweight polypropylene mesh with resorbable polyglactin component | Improved integration with lower weight mesh with reduced inflammation and fibrosis. Heavier weight mesh induced an intense chronic inflammatory reaction with intensive scar bridging formation. This was demonstrated by increased rates of apoptotic and proliferation cells. |
| **Scheibach et al (2004)** | Pig | Groin | 44 | - Heavyweight polypropylene - Lightweight polypropylene - Lightweight polypropylene mesh coated with titanium - Lightweight polypropylene mesh with resorbable polyglactic acid | Both lightweight polypropylene meshes characterized by a more favourable foreign body reaction, with the titanium coating providing an additional advantage in terms of biocompatibility. |
| **Conze et al (2004)** | Rabbit | Abdomen | 45 | Polypropylene meshes of pore size:   - 0.6mm - 2.5mm - 4.0mm | Heavier weight, small pore meshes showed stronger adhesion formation.  Granuloma formation was lowest in larger pore monofilament meshes. |
| **Jerabek et al (2014)** | Rabbit | Abdomen | 21 | Polypropylene mesh of pore size:   - 3.0 x 2.8mm - 1.0 x 0.8mm - 0.6 x 0.5mm | Largest pore mesh demonstrated lowest level of shrinkage.  The width of foreign body granuloma was reduced with increasing pore size. The width of granuloma was statistically significant between mesh types. |
| **Junge et al (2008)** | Rabbit | Abdomen | 20 | - Lightweight polypropylene - Heavyweight polypropylene | Reduced foreign body granulomas with lower weight mesh.  No significant differences between the mesh types regarding apoptotic and proliferation cells. |
| **Feola et al (2013)** | Parous rhesus macaques | Vagina | 45 | - Heavyweight polypropylene - Lightweight polypropylene - Lightweight Polypropylene with resorbable polyglecaprone-25 component | Heaviest weight mesh had the greatest negative impact on both active and passive mechanics. Greater reduction in vaginal contractility and tissue stiffness of the vagina was found. |
| **Liang et al (2013)** | Parous rhesus macaques | Vagina | 50 | - Heavy weight polypropylene - Lightweight polypropylene - Lightweight polypropylene with resorbable polyglecaprone-25 component | Stiffer, higher density mesh had negative impact on vaginal morphology characterised by thinning of the smooth muscle layer, increased cell apoptosis, increased collagenase activity, decreased collagen and elastin content and increased glycosaminoglycan content. |
| **Liang et al (2015)** | Parous rhesus macaque | Vagina | 39 | - Heavy weight polypropylene - Lightweight polypropylene - Lightweight polypropylene with resorbable polyglecaprone-25 component | Heavier, stiffer mesh was associated with increased matrix degradation. Lower weight , stiff mesh had a less negative impact in terms of degradation. |
| **Cobb et al (2006)** | Pig | Abdomen | 18 | - Heavy weight polypropylene - Mid-weight polypropylene - Lightweight polypropylene with resorbable polyglecaprone-25 component | All meshes exhibited burst strengths that were much greater than the burst strength of the native abdominal wall fascia alone |
| **Ozog et al (2011)** | Rabbit | Abdomen | 40 | - Lightweight polypropylene - Lightweight polypropylene with resorbable polyglecaprone component - Lightweight polypropylene sandwiched between two non-porous layers of polyglecaprone and covered on one side with polydiaxonone - Polyvinylidinefluoride | Biomechanical behaviour of lightweight meshes did not mimic that of native controls. The two lightest weigh meshes had unacceptable handling characteristics making implantation difficult in comparison with the heavier constructs. |
| ***Monofilament versus multifilament*** | | | | | |
| **Krause et al (2006)** | rats | Abdomen | 40 | - Type 1 monofilamentous polypropylene - Type 3 multifilamentous polyglactin (resorbable) - Multifilamentous polypropylene - Multifilamentous Polypropylene with multifilamentous polyglactin | Type 3 multifilamentous polypropylene meshes with and without the polyglactin component had a more marked inflammatory cellular response and fibrotic reaction compared to type 1 mesh.  Monofilamentous polypropylene and the fully resorbable polyglactin mesh had minimal fibrotic reaction. |
| **Diaz Godoy et al (2011)** | Rabbit | Abdomen | 30 | - Heavyweight, medium pore size (800um) polypropylene mesh - Medium-weight, large pore (1.5mm) polypropylene mesh - Lightweight, very large pore (3.6 x 2.8mm) polypropylene mesh | Low-weight, very large pore polypropylene meshes proved best in terms of intestinal contamination. |
| **Badiou et al (2011)** | Wister rats | abdomen | 84 | 4 groups of large pore monofilament polypropylene mesh:   - Silver coated, infected with Escherichia coli - Silver coated, non-infected - Non-silver coated, infected with Escherichia coli - Non-silver coated, non-infected | Erosion was significantly higher in the infected than in non-infected silver coated mesh groups. |
| ***Size of mesh*** | | | | | |
| **Manodoro et al (2013)** | Sheep | Vagina and abdomen | 20 | 2 groups of 28-g/m² polypropylene mesh reinforced with polyglecaprone fibres;   - 50 × 50 mm - 35 × 35 mm | Insertion of a 50 × 50 mm mesh led to exposures in 30% of cases. There were no exposures in the 35 × 35 mm implant group. |
| ***The impact of anatomical location*** | | | | | |
| **Hilger et al (2006)** | Rabbit | Vagina and abdomen | 20 | - Human dermis - Porcine dermis - Porcine collagen-coated polypropylene mesh - Autologous fascia | Decrease in ultimate strength was associated with vaginal implantation. |

*PTFE: Polytetrafluoroethylene
